# Assessing Multiplex Tiling PCR Sequencing Approaches for Detecting Genomic Variants of SARS-CoV-2 in Municipal Wastewater

**DOI:** 10.1128/mSystems.01068-21

**Published:** 2021-10-19

**Authors:** Xuan Lin, Melissa Glier, Kevin Kuchinski, Tenysha Ross-Van Mierlo, David McVea, John R. Tyson, Natalie Prystajecky, Ryan M. Ziels

**Affiliations:** a Civil Engineering, The University of British Columbia, Vancouver, British Columbia, Canada; b Environmental Microbiology, British Columbia Center for Disease Control Public Health Laboratory, Vancouver, British Columbia, Canada; c Pathology and Laboratory Medicine, The University of British Columbia, Vancouver, British Columbia, Canada; d Biological Sciences, Simon Fraser University, Burnaby, British Columbia, Canada; e Environmental Health Services, British Columbia Center for Disease Control Public Health Laboratory, Vancouver, British Columbia, Canada; Princeton University

**Keywords:** COVID-19, RNA, SARS-CoV-2, epidemiology, variants, wastewater, whole-genome sequencing

## Abstract

Wastewater-based genomic surveillance of the severe acute respiratory syndrome coronavirus 2 (SARS-CoV-2) virus shows promise to complement genomic epidemiology efforts. Multiplex tiling PCR is a desirable approach for targeted genome sequencing of SARS-CoV-2 in wastewater due to its low cost and rapid turnaround time. However, it is not clear how different multiplex tiling PCR primer schemes or wastewater sample matrices impact the resulting SARS-CoV-2 genome coverage. The objective of this work was to assess the performance of three different multiplex primer schemes, consisting of 150-bp, 400-bp, and 1,200-bp amplicons, as well as two wastewater sample matrices, influent wastewater and primary sludge, for targeted genome sequencing of SARS-CoV-2. Wastewater samples were collected weekly from five municipal wastewater treatment plants (WWTPs) in the Metro Vancouver region of British Columbia, Canada during a period of increased coronavirus disease 19 (COVID-19) case counts from February to April 2021. RNA extracted from clarified influent wastewater provided significantly higher genome coverage (breadth and median depth) than primary sludge samples across all primer schemes. Shorter amplicons appeared to be more resilient to sample RNA degradation but were hindered by greater primer pool complexity in the 150-bp scheme. The identified optimal primer scheme (400 bp) and sample matrix (influent) were capable of detecting the emergence of mutations associated with genomic variants of concern, for which the daily wastewater load significantly correlated with clinical case counts. Taken together, these results provide guidance on best practices for implementing wastewater-based genomic surveillance and demonstrate its ability to inform epidemiology efforts by detecting genomic variants of concern circulating within a geographic region.

**IMPORTANCE** Monitoring the genomic characteristics of the SARS-CoV-2 virus circulating in a population can shed important insights into epidemiological aspects of the COVID-19 outbreak. Sequencing every clinical patient sample in a highly populous area is a difficult feat, and thus sequencing SARS-CoV-2 RNA in municipal wastewater offers great promise to augment genomic surveillance by characterizing a pooled population sample matrix, particularly during an escalating outbreak. Here, we assess different approaches and sample matrices for rapid targeted genome sequencing of SARS-CoV-2 in municipal wastewater. We demonstrate that the optimal approach is capable of detecting the emergence of SARS-CoV-2 genomic variants of concern, with strong correlations to clinical case data in the province of British Columbia. These results provide guidance on best practices on, as well as further support for, the application of wastewater genomic surveillance as a tool to augment current genomic epidemiology efforts.

## OBSERVATION

Genomic surveillance of the severe acute respiratory syndrome coronavirus 2 (SARS-CoV-2) virus plays a critical role in tracking its evolution during the current global coronavirus disease 2019 (COVID-19) pandemic (1–3). Recently, several emerging lineages of SARS-CoV-2, so-called variants of concern (VoCs), have been associated with increased levels of transmission (4), disease severity (5), and/or immune escape (6, 7). These VoCs have originated from various locations globally (4, 8), but they are spreading within new geographic regions due to travel-associated and local transmission (9). Providing rapid detection of VoC infections within a population could thus help to inform effective public health outbreak mitigation strategies.

Since the SARS-CoV-2 virus is shed in feces during infection (10), viral genome fragments can be detected in municipal wastewater and have been associated with clinical case numbers within contributing regions (11–14). Previous work has demonstrated the potential to sequence SARS-CoV-2 fragments in municipal wastewater and detect single-nucleotide variants (SNVs) that correspond to clinical cases in the contributing sewershed (15–17). As SARS-CoV-2 titers in wastewater are relatively low (11, 13), an enrichment step is typically needed prior to sequencing to improve sensitivity (15). The two main approaches for enriching SARS-CoV-2 RNA in wastewater include oligonucleotide-based capture (15) and multiplex tiling PCR-based targeted amplification (16, 17). The latter approach is promising for wastewater-based viral genomic surveillance due to its lower reagent cost and the potential to be deployed rapidly and in remote locations (18). An important consideration for applying multiplex tiling PCR is the average amplicon length, as this can impact assay sensitivity in the case of RNA degradation (19). This could be particularly important for its application to wastewater-based epidemiology, as SARS-CoV-2 particles and free RNA can undergo variable levels of degradation (20, 21) and may differ based on the type of wastewater sample matrix (e.g., influent versus primary sludge) (22). We therefore hypothesized that there may be an optimal tiling PCR amplicon size and an optimal wastewater sample matrix type that enable adequate genome coverage of SARS-CoV-2 for the identification of genomic VoCs.

### SARS-CoV-2 genome coverage is greater with influent wastewater ultrafiltration than with direct sludge extraction and is impacted by multiplex tiling PCR amplicon length.

**FIG 1:**
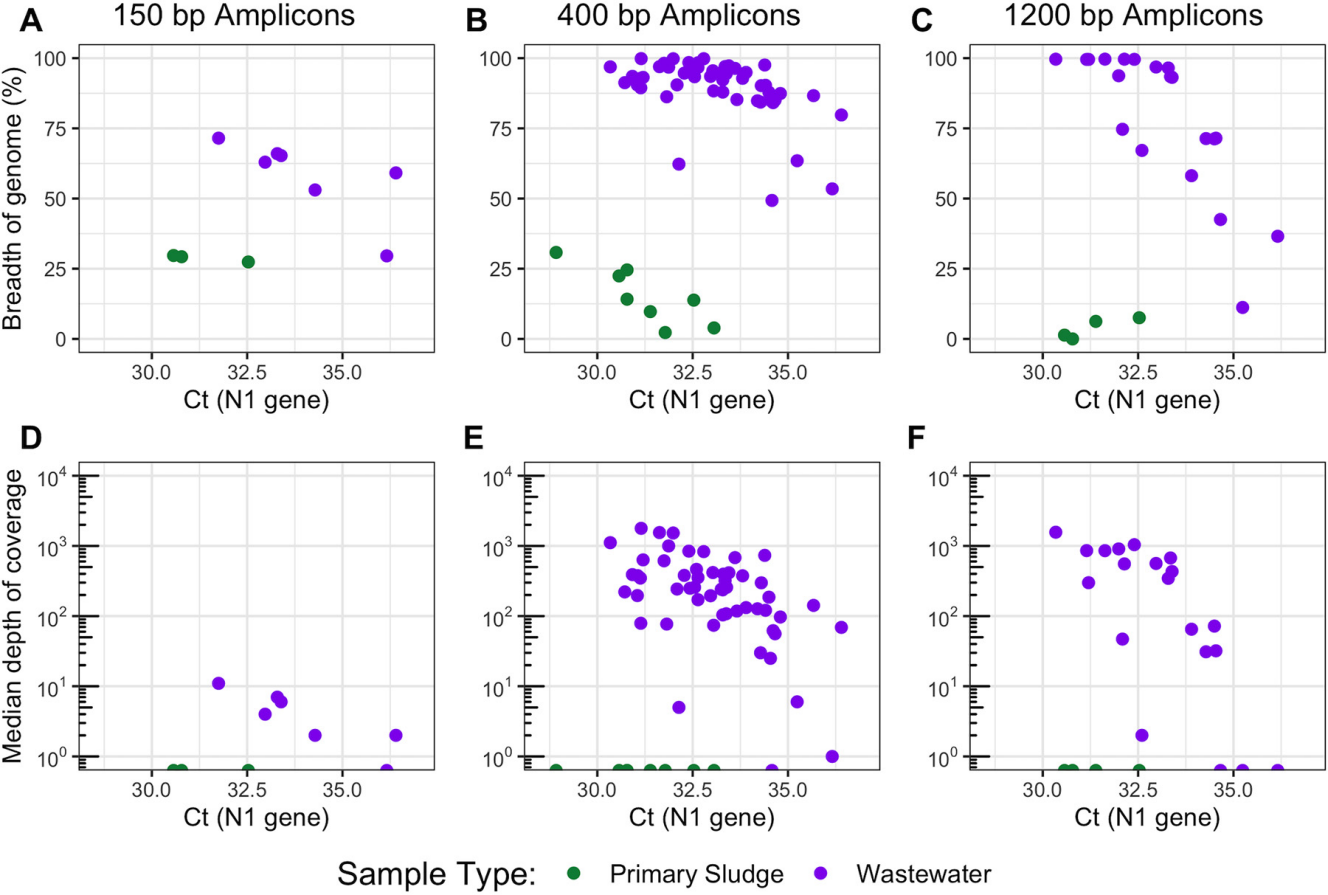
SARS-CoV-2 whole-genome sequencing coverage results from three multiplex tiling PCR primer schemes, including breadth of genome coverage for 150-bp amplicons (A), 400-bp amplicons (B), and 1,200-bp amplicons (C), as well as the median depth of coverage across the genome for 150-bp amplicons (D), 400-bp amplicons (E), and 1,200-bp amplicons (F). The breadth of coverage represents the proportion of nucleotides in the SARS-CoV-2 reference genome (NCBI accession number MN908947.3) that are covered by at least one read, and the median depth of coverage represents the median number of reads mapped at each nucleotide position across the SARS-CoV-2 reference genome. Values are plotted versus the sample cycle threshold (*C_T_*) value for the U.S. CDC N1 assay, measured by reverse transcription-quantitative PCR (RT-qPCR) (see Text S1 in the supplemental material). Data points aligned with the *x* axis (plots D to F) had values of zero and could not be log transformed.

We sequenced a total of 96 wastewater samples collected between 7 February and 18 April 2021 across five municipal WWTPs in Vancouver, Canada, using the following three different primer schemes for multiplex tiling PCR of SARS-CoV-2: Swift Bioscience’s 150-bp amplicon scheme (*n* = 10 total, 3 sludge and 7 influent), the Artic 400-bp amplicon scheme (23) (*n* = 62 total, 8 sludge and 54 influent), and the Freed/midnight 1,200-bp amplicon scheme (24) (*n* = 24 total, 4 sludge and 20 influent) (detailed methods are given in Text S1 in the supplemental material). Sludge samples failed to produce libraries with over 32% breadth of genome coverage across all primer schemes and sample cycle thresholds (*C_T_*) (Fig. 1A to C). Conversely, influent wastewater samples produced libraries that had significantly higher breadth of coverage across all primer schemes (*P < *0.01, Tukey’s test; Fig. 1). One possible explanation for this finding could be that the sludge matrix was inhibitory to reverse transcription-PCR (RT-PCR) (11); however, no inhibition of reverse transcription-quantitative PCR on sludge RNA extracts was detected using internal controls (see Text S1 in the supplemental material and Table S2 at https://doi.org/10.6084/m9.figshare.16416528). Another potential reason for the lower genome coverage in sludge was that SARS-CoV-2 was more nonintact or its RNA was more degraded with the direct sludge extraction compared to ultrafiltration of influent wastewater, as has been previously hypothesized (22). A third potential cause of discrepancies in genome coverage between sludge and influent wastewater samples could be higher off-target amplification in sludge extracts. Correspondingly, sample type significantly impacted the fraction of on-target reads for all schemes after accounting for *C_T_* values (*P < *0.01, two-way analysis of covariance [ANCOVA]), with mean mapping rates of sludge samples being over 100 times lower than that of influent samples (0.01% versus 11.3%, respectively; Table S1). Therefore, ultrafiltration of influent wastewater provided more suitable RNA extracts for multiplexed tiling PCR of SARS-CoV-2 than did direct extraction from wastewater sludge, likely due to a combination of greater SARS-CoV-2 RNA degradation and greater off-target amplification in sludge.

10.1128/mSystems.01068-21.1 TEXT S1 Supplemental methods. Download Text S1, PDF file, 0.1 MB. Copyright © 2021 Lin et al. 2021 Lin et al. https://creativecommons.org/licenses/by/4.0/ This content is distributed under the terms of the Creative Commons Attribution 4.0 International license.

10.1128/mSystems.01068-21.10 TABLE S1 Sample metadata, including sample epidemiological week and sampling date, WWTP, sample type, daily average flow rate, cycle threshold (*C_T_*) of the U.S. CDC N1 assay, sequencing library type, sequencing throughput, mapping rates, and NCBI accession number for raw read data. For sample type, WW indicates influent wastewater and PS indicates primary sludge. Download Table S1, DOCX file, 0.04 MB. Copyright © 2021 Lin et al. 2021 Lin et al. https://creativecommons.org/licenses/by/4.0/ This content is distributed under the terms of the Creative Commons Attribution 4.0 International license.

If the level of RNA degradation within a wastewater sample impacts the resulting SARS-CoV-2 genome coverage, we would expect to see less of a drop-off in coverage at high *C_T_* values for schemes with shorter amplicons. Indeed, we detected a significant effect of amplicon length on the breadth of genome coverage as a function of *C_T_* (*P < *0.01, two-way ANCOVA). The median genome coverage with the 150-bp amplicon scheme spanned one order of magnitude within influent wastewater samples that had *C_T_* values ranging from 31 to 37 (Fig. 1D), while those from the 400-bp and 1,200-bp schemes spanned 3.2 and 3.0 orders of magnitude, respectively (Fig. 1E and F). Improvements with the 400-bp scheme versus the 1,200-bp scheme were marginal, yet 83% of paired influent samples with *C_T_* values over 32.5 (10 of 12) showed higher breadth of coverage with the 400-bp scheme (Fig. S1). Thus, shorter amplicon schemes may be more robust to sample RNA degradation at higher *C_T_* values. However, there was a trade-off between amplicon length and genome coverage, as the magnitudes of the median genome coverage and breadth of coverage obtained with the 150-bp scheme and influent samples were significantly lower than those obtained with the 400-bp scheme (*P = *0.022 and 5.0 × 10^−9^, respectively; Tukey’s test). This result likely cannot be attributed to library preparation and/or the sequencing platform, as we found no significant difference in the breadth of genome coverage obtained on a subset of 10 400-bp amplicon samples sequenced with both Illumina MiSeq and Oxford Nanopore Technologies MinION platforms (*P = *0.5, Tukey’s test; Fig. S2). The lower breadth of coverage observed with the 150-bp scheme could thus have been caused by more primer-primer interactions with a larger number of primers or by the characteristics of the primer pool design (19). Such effects were also indicated by sequencing a synthetic SARS-CoV-2 RNA genome as a positive control, as the 150-bp primer scheme showed more uneven coverage across the genome compared to that of the 400-bp and 1,200-bp schemes (Fig. S3). Therefore, the 400-bp primer scheme appears to strike a balance between resilience to sample RNA degradation and mitigation of issues around primer pool complexity and multiplex amplicon balancing.

10.1128/mSystems.01068-21.2 FIG S1 Breadth of genome coverage for paired samples with cycle threshold (*C_T_*) values of >32.5 sequenced with both the 400-bp and 1,200-bp primer schemes. Points are colored based on the sample *C_T_* value (U.S. CDC N1 gene; see Text S1), and the dotted line indicates a sample pair. Coverage was based on mapping quality-filtered reads to the severe acute respiratory syndrome coronavirus 2 (SARS-CoV-2) Wuhan-Hu-1 reference genome sequence (NCBI accession number MN908947.3). Download FIG S1, PDF file, 0.3 MB. Copyright © 2021 Lin et al. 2021 Lin et al. https://creativecommons.org/licenses/by/4.0/ This content is distributed under the terms of the Creative Commons Attribution 4.0 International license.

10.1128/mSystems.01068-21.3 FIG S2 Breadth of SARS-CoV-2 genome coverage obtained with 10 samples amplified with the 400-bp primer scheme and sequenced on an Illumina MiSeq instrument in 2 × 250-bp mode or on an ONT MinION instrument, according to the library preparation outlined in Text S1. The 10 samples that were selected for this paired comparison are listed in Table S1. Points are colored based on the sample *C_T_* value (U.S. CDC N1 gene; see Text S1), and the dotted line indicates a sample pair. Coverage was based on mapping quality-filtered reads to the SARS-CoV-2 Wuhan-Hu-1 reference genome sequence (NCBI accession number MN908947.3). Download FIG S2, PDF file, 0.1 MB. Copyright © 2021 Lin et al. 2021 Lin et al. https://creativecommons.org/licenses/by/4.0/ This content is distributed under the terms of the Creative Commons Attribution 4.0 International license.

10.1128/mSystems.01068-21.4 FIG S3 Depth of genome coverage across the SARS-CoV-2 genome for samples of a synthetic RNA genome control (102019, Twist Control-1; Twist Biosciences), prepared and sequenced with the 150-bp primer scheme on an Illumina MiSeq instrument in 2 × 150-bp mode (A), the 400-bp primer scheme on an Illumina MiSeq instrument in 2 × 250-bp mode (B), the 400-bp primer scheme on an ONT MinION instrument (C), and the 1,200-bp primer scheme on an ONT MinION instrument (D). These synthetic RNA genome samples were included as positive controls in all sequencing runs. Coverage depth was determined by mapping quality-filtered reads to the SARS-CoV-2 synthetic control genome sequence (GenBank accession number MT007544.1). Note that the Twist Control-1 synthetic genome has breaks approximately every 5 kb, which is the reason for amplicon dropout in those regions. Download FIG S3, PDF file, 0.4 MB. Copyright © 2021 Lin et al. 2021 Lin et al. https://creativecommons.org/licenses/by/4.0/ This content is distributed under the terms of the Creative Commons Attribution 4.0 International license.

### SARS-CoV-2 whole-genome sequencing from wastewater captures the emergence of genomic variants in a geographic region.

**FIG 2:**
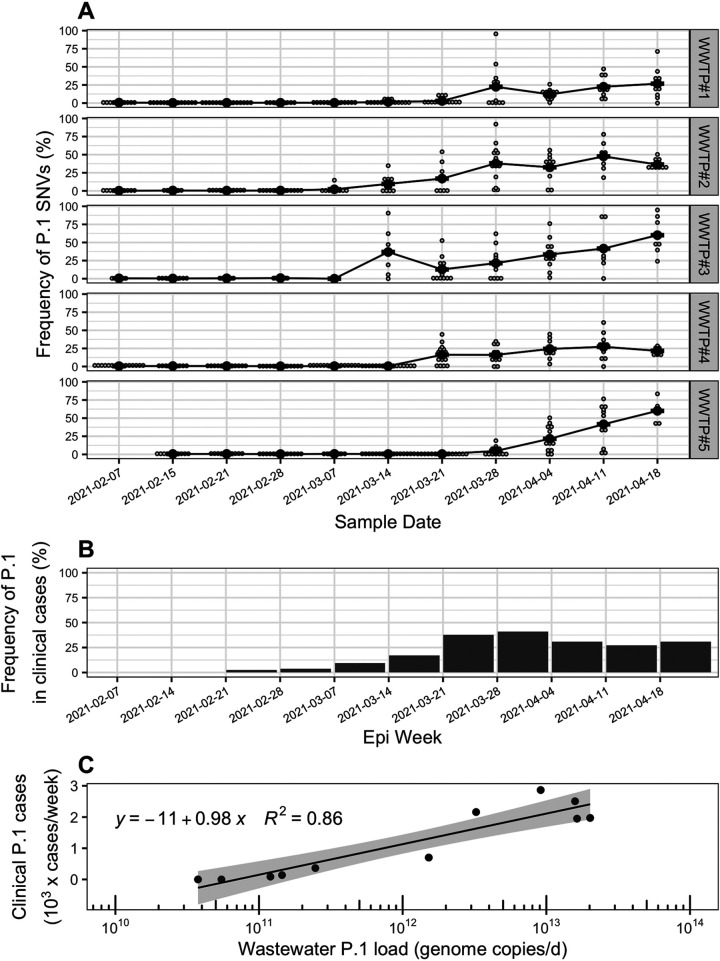
(A) Frequency of single-nucleotide variants (SNVs) associated with the P.1 lineage of SARS-CoV-2 within influent wastewater samples from five wastewater treatment plants in Vancouver, British Columbia (BC), from 7 February to 18 April 2021. Smaller gray dots represent the frequency of individual variant of concern (VoC)-associated SNVs on the sample dates, while the larger black points represent the mean across all detected VoC-associated SNVs. Only genome positions with a read coverage over 50 are included in SNV frequency calculations. The VoC-associated SNVs are described in Text S1 in the supplemental material and provided in Table S3 at https://doi.org/10.6084/m9.figshare.16416528. (B) Frequency of the P.1 lineage in clinical COVID-19 patient cases in the province of BC, Canada, over the study period. The frequencies in clinical patient cases correspond to average values detected over an epidemiology (epi) week and were adapted from reference 25. (C) Correlation between the wastewater cumulative daily load of P.1 genomes summed across all five wastewater treatment plants (WWTPs) and the total P.1 clinical cases in the province of BC observed within the same epidemiological week. The wastewater P.1 daily load (genome copies/day) was approximated by normalizing copies of the SARS-CoV-2 N1 gene (copies/liter) by daily flow rates (liters/day) to obtain N1 loads (copies/day) for all WWTPs and multiplying those by the mean frequency of P.1-associated SNVs in each WWTP across all sample dates. For each date, the cumulative P.1 daily load was determined by summing the P.1 loads across all five WWTPs. The P.1 clinical case counts by week were estimated from reference 25 by multiplying total provincial COVID-19 case counts by the frequency of P.1 in clinical provincial cases.

The sequence data produced via the 400-bp primer scheme and influent wastewater samples was used to measure the frequency of VoC-associated SNVs (see Table S3 at https://doi.org/10.6084/m9.figshare.16416528) across the five WWTPs over the study period. SNVs associated with the VoC lineages B.1.1.7 and P.1 both increased to a maximum mean frequency of 60% across all WWTPs (Fig. 2A and Fig. S4 to S6), while the frequency of B.1.351 did not substantially increase (Fig. S7). These findings align with the results of clinical screening and sequencing of patient samples over the same period within the province of British Columbia, during which P.1 and B.1.1.7 became the dominant lineages, while B.1.351 did not appreciably spread (25) (Fig. 2B and Fig. S5 and S7). At the time of publishing, VoC frequency data for clinical cases was only available at the provincial level, yet the health service areas corresponding to the 5 WWTP sewersheds accounted for 74% of total cases in the province during the study period (25). The log-transformed, flow-normalized daily loads of P.1 and B.1.1.7 across all WWTPs (in genome copies/day) were strongly correlated with clinical case counts of those lineages within the province for the corresponding epidemiological weeks (*R*^2^ = 0.86 and 0.85, respectively; Fig. 2C and Fig. S5). The log-transformed mean frequencies of VoC-associated SNVs in wastewater were also significantly correlated with that of VoC clinical case counts within the region (*P* < 0.01; Fig. S8). Therefore, the frequency of VoC-associated SNVs within influent wastewater measured with multiplex tiling PCR is a suitable measure to monitor community transmission of genomic variants within a sewershed. The onset of P.1- and B.1.1.7-associated SNVs within influent wastewater followed different patterns for the five WWTPs, providing additional support that wastewater SARS-CoV-2 sequencing can illuminate localized spread of genomic variants on a regional scale (15, 17). The rapid turnaround time (here, ∼3 days from sampling to data generation), low capital cost, and high portability of nanopore sequencing combined with highly multiplexed tiling PCR for SARS-CoV-2 sequencing of wastewater shows great promise to complement genomic epidemiology efforts during the COVID-19 pandemic by detecting the emergence of VoC within a pooled population sample.

10.1128/mSystems.01068-21.5 FIG S4 Heatmap showing the frequencies of single nucleotide variants (SNVs) associated with the P.1 lineage over the study period in the influent wastewater of five municipal wastewater treatment plants (WWTPs) in Metro Vancouver, British Columbia. Values are shown only for SNVs detected with a total read coverage of 50 or greater at that genomic position, and otherwise the value is shown as white. SNVs that never had a read coverage over 50 across all samples were filtered. All P.1-associated SNV sites that were queried are provided in Table S3 at https://doi.org/10.6084/m9.figshare.16416528. Download FIG S4, PDF file, 0.01 MB. Copyright © 2021 Lin et al. 2021 Lin et al. https://creativecommons.org/licenses/by/4.0/ This content is distributed under the terms of the Creative Commons Attribution 4.0 International license.

10.1128/mSystems.01068-21.6 FIG S5 (A) Frequency of SNVs associated with the B.1.1.7 lineage of SARS-CoV-2 within influent wastewater samples from five wastewater treatment plants in Metro Vancouver, British Columbia (BC), from 7 February to 18 April 18 2021. Smaller grey dots represent the frequency of individual SNVs, while the larger black points represent the mean frequency across all detected SNVs. Only genome positions with a read coverage over 50 are included in SNV frequency calculations. (B) Frequency of the B.1.1.7 lineage in clinical coronavirus disease 2019 (COVID-19) patient cases in the province of BC, Canada, over the study period. Frequencies in clinical patient cases correspond to average values detected over an epidemiology (epi) week, and were adapted from reference 25. (C) Correlation between the wastewater cumulative daily load of B.1.1.7 genomes summed across all five WWTPs and the total B.1.1.7 clinical cases in the province of BC observed within the same epidemiological week. The wastewater B.1.1.7 daily load (genome copies/day) was approximated by normalizing copies of the N1 gene (copies/liter) by daily flow rates (liters/day) to obtain N1 loads (copies/day) for all WWTPs and multiplying those by the mean frequency of B.1.1.7-associated SNVs in each WWTP across all sample dates. For each date, the cumulative B.1.1.7 daily load was determined by summing the B.1.1.7 loads across all five WWTPs. The B.1.1.7 clinical case counts were adapted from reference 25 by multiplying total provincial COVID-19 case counts by the frequency of B.1.1.7 in clinical provincial cases. Download FIG S5, PDF file, 0.4 MB. Copyright © 2021 Lin et al. 2021 Lin et al. https://creativecommons.org/licenses/by/4.0/ This content is distributed under the terms of the Creative Commons Attribution 4.0 International license.

10.1128/mSystems.01068-21.7 FIG S6 Heatmap showing the frequencies of SNVs associated with the B.1.1.7 lineage over the study period in the influent wastewater from five municipal WWTPs in Metro Vancouver, British Columbia. Values are shown only for SNVs detected with a total read coverage of 50 or greater at that genomic position, and otherwise the value is shown as white. SNVs that never had a read coverage over 50 across all samples were filtered. All B.1.1.7-associated SNV sites that were queried are provided in Table S3 at https://doi.org/10.6084/m9.figshare.16416528. Download FIG S6, PDF file, 0.01 MB. Copyright © 2021 Lin et al. 2021 Lin et al. https://creativecommons.org/licenses/by/4.0/ This content is distributed under the terms of the Creative Commons Attribution 4.0 International license.

10.1128/mSystems.01068-21.8 FIG S7 (A) Frequency of SNVs associated with the B.1.351 lineage of SARS-CoV-2 within influent wastewater samples from five wastewater treatment plants in Metro Vancouver, British Columbia (BC), from 7 February to 18 April 2021. Smaller grey dots represent the frequency of individual SNVs, while the larger black points represent means across all detected SNVs. Only genome positions with a read coverage over 50 are included in SNV frequency calculations. (B) Frequency of the B.1.351 lineage in clinical COVID-19 patient cases in the province of BC, Canada, over the study period. Frequencies in clinical patient cases correspond to an average value detected over an epidemiology (epi) week and were adapted from reference 25. Download FIG S7, PDF file, 0.5 MB. Copyright © 2021 Lin et al. 2021 Lin et al. https://creativecommons.org/licenses/by/4.0/ This content is distributed under the terms of the Creative Commons Attribution 4.0 International license.

10.1128/mSystems.01068-21.9 FIG S8 Correlation between the frequency of variant of concern (VoC)-associated clinical cases in the province of British Columbia (log_10_ transformed) and the weighted-mean frequency of VoC-associated SNVs across the 5 WWTPs within the same epidemiological week (log_10_ transformed), shown for the VoCs P.1 (A) and B.1.1.7 (B). The wastewater VoC-associated SNV frequencies were calculated as a mean of those frequencies within the individual WWTPs weighted by their respective WWTP daily load of the SARS-CoV-2 N1 gene (genome copies/day). The VoC-associated frequencies of clinical case counts by week were adapted from reference 25. Data points with zero clinical cases are shown aligned to the *x* axis. Download FIG S8, PDF file, 0.01 MB. Copyright © 2021 Lin et al. 2021 Lin et al. https://creativecommons.org/licenses/by/4.0/ This content is distributed under the terms of the Creative Commons Attribution 4.0 International license.

### Data availability.

The raw reads associated with all samples are available in the Sequence Read Archive under BioProject accession number PRJNA731975. The accession numbers for each sample are also provided in Table S1 in the supplemental material, along with the sample metadata.
