# Supplementary material for: Assessing Multiplex Tiling PCR Sequencing Approaches for Detecting Genomic Variants of SARS-CoV-2 in Municipal Wastewater
Source: mSystems. 2021 Oct 19;6(5):e01068-21. doi: 10.1128/mSystems.01068-21 (PMC8525555; doi:10.1128/mSystems.01068-21)
Supplement: TABLE S1 [file msystems.01068-21-st001.docx]

| **Epi Week** | **Date sampled** | **WWTP** | **Sample type** | **Flow rate (MLD)** | **US CDC N1 RT-qPCR** | | **Amplicon size (bp)** | **Amplicon concentration (post PCR) (ng/μL)** | **Sequencing Platform** | **Raw bases (bp)** | **Bases mapped (bp)** | **Mapping rate** | **NCBI accession number** |
| --- | --- | --- | --- | --- | --- | --- | --- | --- | --- | --- | --- | --- | --- |
|  |  |  |  |  | **C_t_** | **Quantity (copies/L influent or copies/g-TS sludge)** |  |  |  |  |  |  |  |
| 5 | 07-Feb-21 | WWTP#1 | WW | 596 | 33.66 | 6.81E+03 | 400 | 13.2 | ONT_MinION | 8.35E+08 | 1.86E+07 | 2.2% | SAMN19321363 |
| 5 | 07-Feb-21 | WWTP#1 | PS | N/A | 31.78 | N/A | 400 | 25.4 | ONT_MinION | 4.78E+07 | 7.61E+02 | 0.0% | SAMN19321367 |
| 5 | 07-Feb-21 | WWTP#2 | WW | 527 | 34.20 | 4.67E+03 | 400 | 28.0 | ONT_MinION | 1.09E+08 | 1.19E+07 | 10.9% | SAMN19321364 |
| 5 | 07-Feb-21 | WWTP#3 | WW | 90 | 34.54 | 3.74E+03 | 400 | 54.4 | ONT_MinION | 1.62E+08 | 3.62E+06 | 2.2% | SAMN19304050 |
| 5 | 07-Feb-21 | WWTP#3 | WW | 90 | 34.54 | 3.74E+03 | 1200 | 26.6 | ONT_MinION | 4.33E+08 | 4.43E+06 | 1.0% | SAMN19304105 |
| 5 | 07-Feb-21 | WWTP#4 | WW | 72 | 33.61 | 6.97E+03 | 400 | 22.0 | ONT_MinION | 5.47E+08 | 5.34E+07 | 9.7% | SAMN19321365 |
| 6 | 15-Feb-21 | WWTP#1 | WW | 593 | 32.60 | 1.38E+04 | 400 | 52.4 | ONT_MinION | 3.63E+08 | 3.11E+07 | 8.6% | SAMN19304051 |
| 6 | 15-Feb-21 | WWTP#1 | PS | N/A | 30.78 | N/A | 400 | 27.0 | ONT_MinION | 1.25E+08 | 6.45E+05 | 0.5% | SAMN19321368 |
| 6 | 15-Feb-21 | WWTP#1 | WW | 593 | 32.60 | 1.38E+04 | 1200 | 26.8 | ONT_MinION | 6.47E+08 | 3.86E+06 | 0.6% | SAMN19304106 |
| 6 | 15-Feb-21 | WWTP#2 | WW | 997 | 34.28 | 4.38E+03 | 150 | 27.2 | Illumina_MiSeq_2x150 | 4.51E+08 | 7.10E+06 | 1.6% | SAMN19304040 |
| 6 | 15-Feb-21 | WWTP#2 | WW | 997 | 34.28 | 4.38E+03 | 400 | 55.8 | ONT_MinION | 6.63E+07 | 3.26E+06 | 4.9% | SAMN19304052 |
| 6 | 15-Feb-21 | WWTP#2 | WW | 997 | 34.28 | 4.38E+03 | 400 | 55.8 | Illumina_MiSeq_2x250 | 8.64E+08 | 3.21E+07 | 3.7% | SAMN20804814 |
| 6 | 15-Feb-21 | WWTP#2 | WW | 997 | 34.28 | 4.38E+03 | 1200 | 28.2 | ONT_MinION | 4.02E+08 | 8.30E+06 | 2.1% | SAMN19304107 |
| 6 | 15-Feb-21 | WWTP#3 | WW | 88 | 35.67 | 1.84E+03 | 400 | 26.4 | ONT_MinION | 2.12E+08 | 1.39E+07 | 6.5% | SAMN19321366 |
| 6 | 15-Feb-21 | WWTP#4 | WW | 76 | 34.41 | 4.23E+03 | 400 | 53.6 | ONT_MinION | 2.71E+08 | 1.10E+07 | 4.1% | SAMN19304053 |
| 6 | 15-Feb-21 | WWTP#5 | WW | 15 | 34.80 | 3.14E+03 | 400 | 50.8 | ONT_MinION | 3.68E+08 | 9.32E+06 | 2.5% | SAMN19304054 |
| 6 | 15-Feb-21 | WWTP#5 | WW | 15 | 34.80 | 3.14E+03 | 400 | 50.8 | Illumina_MiSeq_2x250 | 3.65E+08 | 1.57E+07 | 4.3% | SAMN20804823 |
| 7 | 21-Feb-21 | WWTP#1 | WW | 600 | 31.75 | 2.73E+04 | 150 | 27.4 | Illumina_MiSeq_2x150 | 4.63E+08 | 2.38E+07 | 5.2% | SAMN19304041 |
| 7 | 21-Feb-21 | WWTP#1 | WW | 600 | 31.75 | 2.73E+04 | 400 | 38.2 | ONT_MinION | 3.80E+08 | 1.50E+07 | 4.0% | SAMN19304055 |
| 7 | 21-Feb-21 | WWTP#1 | WW | 600 | 31.75 | 2.73E+04 | 400 | 38.2 | Illumina_MiSeq_2x250 | 5.67E+08 | 6.95E+07 | 12.3% | SAMN20804820 |
| 7 | 21-Feb-21 | WWTP#1 | PS | N/A | 33.06 | N/A | 400 | 23.2 | ONT_MinION | 6.45E+07 | 3.92E+03 | 0.0% | SAMN19321369 |
| 7 | 21-Feb-21 | WWTP#2 | WW | 626 | 32.64 | 1.50E+04 | 400 | 48.8 | ONT_MinION | 9.27E+07 | 2.80E+06 | 3.0% | SAMN19304056 |
| 7 | 21-Feb-21 | WWTP#3 | WW | 93 | 35.24 | 2.89E+03 | 400 | 44.6 | ONT_MinION | 1.33E+08 | 1.31E+06 | 1.0% | SAMN19304057 |
| 7 | 21-Feb-21 | WWTP#3 | WW | 93 | 35.24 | 2.89E+03 | 1200 | 21.0 | ONT_MinION | 3.93E+08 | 1.06E+06 | 0.3% | SAMN19304108 |
| 7 | 21-Feb-21 | WWTP#4 | WW | 71 | 32.43 | 1.72E+04 | 400 | 40.0 | ONT_MinION | 3.68E+08 | 1.04E+07 | 2.8% | SAMN19304058 |
| 7 | 21-Feb-21 | WWTP#5 | WW | 17 | 33.90 | 7.09E+03 | 400 | 39.2 | ONT_MinION | 4.29E+08 | 5.79E+06 | 1.4% | SAMN19304059 |
| 7 | 21-Feb-21 | WWTP#5 | WW | 17 | 33.90 | 7.09E+03 | 1200 | 19.8 | ONT_MinION | 4.32E+08 | 1.45E+07 | 3.3% | SAMN19304109 |
| 8 | 28-Feb-21 | WWTP#1 | PS | N/A | 30.57 | 1.32E+05 | 150 | 25.0 | Illumina_MiSeq_2x150 | 3.74E+08 | 8.77E+05 | 0.2% | SAMN19304042 |
| 8 | 28-Feb-21 | WWTP#1 | WW | 565 | 32.14 | 3.41E+04 | 400 | 10.3 | ONT_MinION | 3.65E+08 | 7.82E+06 | 2.1% | SAMN19304061 |
| 8 | 28-Feb-21 | WWTP#1 | PS | N/A | 30.57 | 1.32E+05 | 400 | 29.8 | ONT_MinION | 1.58E+08 | 6.68E+05 | 0.4% | SAMN19304060 |
| 8 | 28-Feb-21 | WWTP#1 | WW | 565 | 32.14 | 3.41E+04 | 1200 | 16.0 | ONT_MinION | 7.15E+08 | 5.27E+07 | 7.4% | SAMN19304111 |
| 8 | 28-Feb-21 | WWTP#1 | PS | N/A | 30.57 | 1.32E+05 | 1200 | 18.7 | ONT_MinION | 3.41E+08 | 4.06E+02 | 0.0% | SAMN19304110 |
| 8 | 28-Feb-21 | WWTP#2 | WW | 639 | 33.25 | 1.80E+04 | 400 | 29.6 | ONT_MinION | 3.43E+08 | 9.00E+06 | 2.6% | SAMN19304062 |
| 8 | 28-Feb-21 | WWTP#3 | WW | 83 | 34.58 | 6.59E+03 | 400 | 36.6 | ONT_MinION | 3.11E+08 | 8.28E+05 | 0.3% | SAMN19304063 |
| 8 | 28-Feb-21 | WWTP#4 | WW | 70 | 33.38 | 1.53E+04 | 400 | 33.2 | ONT_MinION | 4.10E+08 | 3.25E+06 | 0.8% | SAMN19304064 |
| 8 | 28-Feb-21 | WWTP#5 | WW | 16 | 32.64 | 2.44E+04 | 400 | 34.4 | ONT_MinION | 4.74E+08 | 1.11E+07 | 2.4% | SAMN19304065 |
| 9 | 07-Mar-21 | WWTP#1 | PS | N/A | 30.78 | 8.22E+04 | 150 | 30.8 | Illumina_MiSeq_2x150 | 4.08E+08 | 3.01E+05 | 0.1% | SAMN19304043 |
| 9 | 07-Mar-21 | WWTP#1 | WW | 555 | 32.40 | 2.85E+04 | 400 | 38.8 | ONT_MinION | 3.65E+08 | 6.10E+07 | 16.7% | SAMN19304068 |
| 9 | 07-Mar-21 | WWTP#1 | PS | N/A | 30.78 | 8.22E+04 | 400 | 38.0 | ONT_MinION | 1.72E+08 | 5.48E+04 | 0.0% | SAMN19304066 |
| 9 | 07-Mar-21 | WWTP#1 | WW | 555 | 32.40 | 2.85E+04 | 1200 | 30.6 | ONT_MinION | 4.91E+08 | 7.92E+07 | 16.1% | SAMN19304114 |
| 9 | 07-Mar-21 | WWTP#1 | PS | N/A | 30.78 | 8.22E+04 | 1200 | 17.4 | ONT_MinION | 3.94E+08 | 0.00E+00 | 0.0% | SAMN19304112 |
| 9 | 07-Mar-21 | WWTP#2 | WW | 532 | 33.39 | 1.47E+04 | 150 | 33.4 | Illumina_MiSeq_2x150 | 4.35E+08 | 1.22E+07 | 2.8% | SAMN19304044 |
| 9 | 07-Mar-21 | WWTP#2 | WW | 532 | 33.39 | 1.47E+04 | 400 | 51.2 | ONT_MinION | 2.34E+08 | 2.05E+07 | 8.8% | SAMN19304069 |
| 9 | 07-Mar-21 | WWTP#2 | WW | 532 | 33.39 | 1.47E+04 | 400 | 51.2 | Illumina_MiSeq_2x250 | 5.12E+08 | 4.00E+07 | 7.8% | SAMN20804815 |
| 9 | 07-Mar-21 | WWTP#2 | PS | N/A | 31.39 | 5.32E+04 | 400 | 25.0 | ONT_MinION | 2.02E+08 | 4.86E+04 | 0.0% | SAMN19304067 |
| 9 | 07-Mar-21 | WWTP#2 | WW | 532 | 33.39 | 1.47E+04 | 1200 | 25.4 | ONT_MinION | 5.21E+08 | 4.56E+07 | 8.7% | SAMN19304115 |
| 9 | 07-Mar-21 | WWTP#2 | PS | N/A | 31.39 | 5.32E+04 | 1200 | 13.4 | ONT_MinION | 2.48E+08 | 1.15E+04 | 0.0% | SAMN19304113 |
| 9 | 07-Mar-21 | WWTP#3 | WW | 87 | 36.16 | 2.52E+03 | 150 | 32.0 | Illumina_MiSeq_2x150 | 1.48E+08 | 5.62E+05 | 0.4% | SAMN19304045 |
| 9 | 07-Mar-21 | WWTP#3 | WW | 87 | 36.16 | 2.52E+03 | 400 | 45.4 | ONT_MinION | 2.44E+08 | 1.69E+06 | 0.7% | SAMN19304070 |
| 9 | 07-Mar-21 | WWTP#3 | WW | 87 | 36.16 | 2.52E+03 | 400 | 45.4 | Illumina_MiSeq_2x250 | 5.58E+08 | 4.40E+06 | 0.8% | SAMN20804818 |
| 9 | 07-Mar-21 | WWTP#3 | WW | 87 | 36.16 | 2.52E+03 | 1200 | 33.4 | ONT_MinION | 3.98E+08 | 2.61E+06 | 0.7% | SAMN19304116 |
| 9 | 07-Mar-21 | WWTP#4 | WW | 69 | 33.35 | 1.51E+04 | 400 | 35.2 | ONT_MinION | 3.52E+08 | 2.09E+07 | 5.9% | SAMN19304071 |
| 9 | 07-Mar-21 | WWTP#4 | WW | 69 | 33.35 | 1.51E+04 | 1200 | 19.7 | ONT_MinION | 6.26E+08 | 5.80E+07 | 9.3% | SAMN19304117 |
| 9 | 07-Mar-21 | WWTP#5 | WW | 16 | 36.40 | 1.89E+03 | 150 | 25.6 | Illumina_MiSeq_2x150 | 3.77E+08 | 5.04E+06 | 1.3% | SAMN19304046 |
| 9 | 07-Mar-21 | WWTP#5 | WW | 16 | 36.40 | 1.89E+03 | 400 | 32.0 | ONT_MinION | 4.48E+08 | 8.64E+06 | 1.9% | SAMN19304072 |
| 9 | 07-Mar-21 | WWTP#5 | WW | 16 | 36.40 | 1.89E+03 | 400 | 32 | Illumina_MiSeq_2x250 | 3.89E+08 | 1.34E+07 | 3.5% | SAMN20804819 |
| 10 | 14-Mar-21 | WWTP#1 | PS | N/A | 32.53 | 4.11E+04 | 150 | 22.2 | Illumina_MiSeq_2x150 | 3.69E+08 | 3.03E+05 | 0.1% | SAMN19304047 |
| 10 | 14-Mar-21 | WWTP#1 | WW | 488 | 31.15 | 6.67E+04 | 400 | 46.6 | ONT_MinION | 4.02E+08 | 1.10E+08 | 27.5% | SAMN19304075 |
| 10 | 14-Mar-21 | WWTP#1 | PS | N/A | 32.53 | 4.11E+04 | 400 | 36.6 | ONT_MinION | 1.60E+08 | 4.31E+04 | 0.0% | SAMN19304073 |
| 10 | 14-Mar-21 | WWTP#1 | WW | 488 | 31.15 | 6.67E+04 | 1200 | 26.6 | ONT_MinION | 7.30E+08 | 9.31E+07 | 12.8% | SAMN19304119 |
| 10 | 14-Mar-21 | WWTP#1 | PS | N/A | 32.53 | 4.11E+04 | 1200 | 19.9 | ONT_MinION | 2.95E+08 | 1.56E+04 | 0.0% | SAMN19304118 |
| 10 | 14-Mar-21 | WWTP#2 | WW | 444 | 32.97 | 1.98E+04 | 150 | 22.2 | Illumina_MiSeq_2x150 | 3.90E+08 | 1.31E+07 | 3.4% | SAMN19304048 |
| 10 | 14-Mar-21 | WWTP#2 | WW | 444 | 32.97 | 1.98E+04 | 400 | 33.2 | ONT_MinION | 1.18E+08 | 1.61E+07 | 13.6% | SAMN19304076 |
| 10 | 14-Mar-21 | WWTP#2 | WW | 444 | 32.97 | 1.98E+04 | 400 | 33.2 | Illumina_MiSeq_2x250 | 4.53E+08 | 2.70E+07 | 6.0% | SAMN20804816 |
| 10 | 14-Mar-21 | WWTP#2 | PS | N/A | 28.92 | 3.44E+05 | 400 | 42.4 | ONT_MinION | 2.78E+08 | 1.70E+05 | 0.1% | SAMN19304074 |
| 10 | 14-Mar-21 | WWTP#2 | WW | 444 | 32.97 | 1.98E+04 | 1200 | 17.9 | ONT_MinION | 8.00E+08 | 5.17E+07 | 6.5% | SAMN19304120 |
| 10 | 14-Mar-21 | WWTP#3 | WW | 74 | 34.66 | 6.21E+03 | 400 | 38.0 | ONT_MinION | 1.95E+08 | 8.80E+06 | 4.5% | SAMN19304077 |
| 10 | 14-Mar-21 | WWTP#3 | WW | 74 | 34.66 | 6.21E+03 | 1200 | 17.1 | ONT_MinION | 8.16E+08 | 1.06E+07 | 1.3% | SAMN19304121 |
| 10 | 14-Mar-21 | WWTP#4 | WW | 69 | 31.87 | 4.19E+04 | 400 | 35.8 | ONT_MinION | 4.18E+08 | 8.08E+07 | 19.3% | SAMN19304078 |
| 10 | 14-Mar-21 | WWTP#5 | WW | 15 | 32.79 | 2.33E+04 | 400 | 32.6 | ONT_MinION | 4.45E+08 | 5.79E+07 | 13.0% | SAMN19304079 |
| 11 | 21-Mar-21 | WWTP#1 | WW | 544 | 31.63 | 4.81E+04 | 400 | 32.2 | ONT_MinION | 3.08E+08 | 1.12E+08 | 36.5% | SAMN19304080 |
| 11 | 21-Mar-21 | WWTP#1 | WW | 544 | 31.63 | 4.81E+04 | 1200 | 23.0 | ONT_MinION | 4.88E+08 | 5.97E+07 | 12.2% | SAMN19304122 |
| 11 | 21-Mar-21 | WWTP#2 | WW | 686 | 33.29 | 1.59E+04 | 150 | 39.2 | Illumina_MiSeq_2x150 | 4.46E+08 | 2.09E+07 | 4.7% | SAMN19304049 |
| 11 | 21-Mar-21 | WWTP#2 | WW | 686 | 33.29 | 1.59E+04 | 400 | 39.4 | ONT_MinION | 7.45E+07 | 2.43E+07 | 32.6% | SAMN19304081 |
| 11 | 21-Mar-21 | WWTP#2 | WW | 686 | 33.29 | 1.59E+04 | 400 | 39.4 | Illumina_MiSeq_2x250 | 4.78E+08 | 7.72E+07 | 16.2% | SAMN20804817 |
| 11 | 21-Mar-21 | WWTP#2 | WW | 686 | 33.29 | 1.59E+04 | 1200 | 18.7 | ONT_MinION | 4.77E+08 | 5.21E+07 | 10.9% | SAMN19304123 |
| 11 | 21-Mar-21 | WWTP#3 | WW | 95 | 33.31 | 1.59E+04 | 400 | 43.4 | ONT_MinION | 1.40E+08 | 3.65E+07 | 26.0% | SAMN19304082 |
| 11 | 21-Mar-21 | WWTP#4 | WW | 66 | 31.99 | 3.97E+04 | 400 | 32.0 | ONT_MinION | 3.35E+08 | 9.36E+07 | 27.9% | SAMN19304083 |
| 11 | 21-Mar-21 | WWTP#4 | WW | 66 | 31.99 | 3.97E+04 | 1200 | 17.3 | ONT_MinION | 5.27E+08 | 5.31E+07 | 10.1% | SAMN19304124 |
| 11 | 21-Mar-21 | WWTP#5 | WW | 16 | 34.39 | 9.00E+03 | 400 | 30.0 | ONT_MinION | 3.43E+08 | 8.08E+07 | 23.6% | SAMN19304084 |
| 12 | 28-Mar-21 | WWTP#1 | WW | 584 | 32.09 | 3.56E+04 | 400 | 41.2 | ONT_MinION | 1.65E+08 | 2.41E+07 | 14.7% | SAMN19304085 |
| 12 | 28-Mar-21 | WWTP#1 | WW | 584 | 32.09 | 3.56E+04 | 400 | 41.2 | Illumina_MiSeq_2x250 | 4.53E+08 | 8.03E+07 | 17.7% | SAMN20804821 |
| 12 | 28-Mar-21 | WWTP#1 | WW | 584 | 32.09 | 3.56E+04 | 1200 | 34.6 | ONT_MinION | 4.44E+08 | 1.82E+07 | 4.1% | SAMN19304125 |
| 12 | 28-Mar-21 | WWTP#2 | WW | 566 | 33.03 | 1.86E+04 | 400 | 35.2 | ONT_MinION | 3.21E+08 | 3.06E+07 | 9.5% | SAMN19304086 |
| 12 | 28-Mar-21 | WWTP#3 | WW | 86 | 34.50 | 7.40E+03 | 400 | 50.6 | ONT_MinION | 1.93E+08 | 1.65E+07 | 8.5% | SAMN19304087 |
| 12 | 28-Mar-21 | WWTP#3 | WW | 86 | 34.50 | 7.40E+03 | 1200 | 23.8 | ONT_MinION | 5.28E+08 | 1.89E+07 | 3.6% | SAMN19304126 |
| 12 | 28-Mar-21 | WWTP#4 | WW | 65 | 32.28 | 3.09E+04 | 400 | 43.0 | ONT_MinION | 1.12E+08 | 1.97E+07 | 17.7% | SAMN19304088 |
| 12 | 28-Mar-21 | WWTP#5 | WW | 16 | 33.45 | 1.53E+04 | 400 | 47.8 | ONT_MinION | 1.15E+08 | 2.17E+07 | 18.8% | SAMN19304089 |
| 13 | 04-Apr-21 | WWTP#1 | WW | 469 | 30.34 | 1.15E+05 | 400 | 43.6 | ONT_MinION | 2.02E+08 | 7.42E+07 | 36.6% | SAMN19304090 |
| 13 | 04-Apr-21 | WWTP#1 | WW | 469 | 30.34 | 1.15E+05 | 400 | 43.6 | Illumina_MiSeq_2x250 | 4.88E+08 | 3.12E+08 | 63.9% | SAMN20804822 |
| 13 | 04-Apr-21 | WWTP#1 | WW | 469 | 30.34 | 1.15E+05 | 1200 | 24.4 | ONT_MinION | 4.20E+08 | 7.70E+07 | 18.3% | SAMN19304127 |
| 13 | 04-Apr-21 | WWTP#2 | WW | 370 | 31.20 | 6.41E+04 | 400 | 44.8 | ONT_MinION | 2.21E+08 | 6.54E+07 | 29.5% | SAMN19304091 |
| 13 | 04-Apr-21 | WWTP#2 | WW | 370 | 31.20 | 6.41E+04 | 1200 | 28.0 | ONT_MinION | 2.90E+08 | 3.04E+07 | 10.5% | SAMN19304128 |
| 13 | 04-Apr-21 | WWTP#3 | WW | 69 | 34.30 | 7.89E+03 | 400 | 26.8 | ONT_MinION | 1.48E+08 | 3.68E+07 | 24.9% | SAMN19304092 |
| 13 | 04-Apr-21 | WWTP#4 | WW | 64 | 30.72 | 8.90E+04 | 400 | 35.8 | ONT_MinION | 6.31E+07 | 1.82E+07 | 28.8% | SAMN19304093 |
| 13 | 04-Apr-21 | WWTP#5 | WW | 14 | 33.81 | 1.13E+04 | 400 | 25.6 | ONT_MinION | 1.19E+08 | 3.10E+07 | 26.1% | SAMN19304094 |
| 14 | 11-Apr-21 | WWTP#1 | WW | 463 | 31.13 | 6.84E+04 | 400 | 39.4 | ONT_MinION | 3.66E+08 | 8.11E+07 | 22.2% | SAMN19304095 |
| 14 | 11-Apr-21 | WWTP#2 | WW | 364 | 31.82 | 4.23E+04 | 400 | 41.4 | ONT_MinION | 7.10E+07 | 1.96E+07 | 27.6% | SAMN19304096 |
| 14 | 11-Apr-21 | WWTP#3 | WW | 72 | 33.29 | 1.64E+04 | 400 | 32.8 | ONT_MinION | 9.78E+07 | 2.68E+07 | 27.4% | SAMN19304097 |
| 14 | 11-Apr-21 | WWTP#4 | WW | 64 | 31.06 | 7.21E+04 | 400 | 33.8 | ONT_MinION | 3.51E+08 | 9.48E+07 | 27.0% | SAMN19304098 |
| 14 | 11-Apr-21 | WWTP#5 | WW | 14 | 32.55 | 2.58E+04 | 400 | 38.8 | ONT_MinION | 2.91E+08 | 6.91E+07 | 23.7% | SAMN19304099 |
| 15 | 18-Apr-21 | WWTP#1 | WW | 452 | 30.92 | 7.86E+04 | 400 | 41.8 | ONT_MinION | 1.34E+08 | 2.71E+07 | 20.2% | SAMN19304100 |
| 15 | 18-Apr-21 | WWTP#2 | WW | 352 | 31.05 | 7.19E+04 | 400 | 38.2 | ONT_MinION | 1.50E+08 | 2.03E+07 | 13.6% | SAMN19304101 |
| 15 | 18-Apr-21 | WWTP#3 | WW | 67 | 34.61 | 7.20E+03 | 400 | 47.2 | ONT_MinION | 1.11E+08 | 7.30E+06 | 6.5% | SAMN19304102 |
| 15 | 18-Apr-21 | WWTP#4 | WW | 68 | 31.14 | 6.69E+04 | 400 | 51.4 | ONT_MinION | 4.11E+07 | 6.62E+06 | 16.1% | SAMN19304103 |
| 15 | 18-Apr-21 | WWTP#5 | WW | 14 | 33.05 | 1.85E+04 | 400 | 40.6 | ONT_MinION | 4.11E+07 | 5.20E+06 | 12.7% | SAMN19304104 |
