# Supplementary figures and images for: Assessing Multiplex Tiling PCR Sequencing Approaches for Detecting Genomic Variants of SARS-CoV-2 in Municipal Wastewater
Source: mSystems. 2021 Oct 19;6(5):e01068-21. doi: 10.1128/mSystems.01068-21 (PMC8525555; doi:10.1128/mSystems.01068-21)

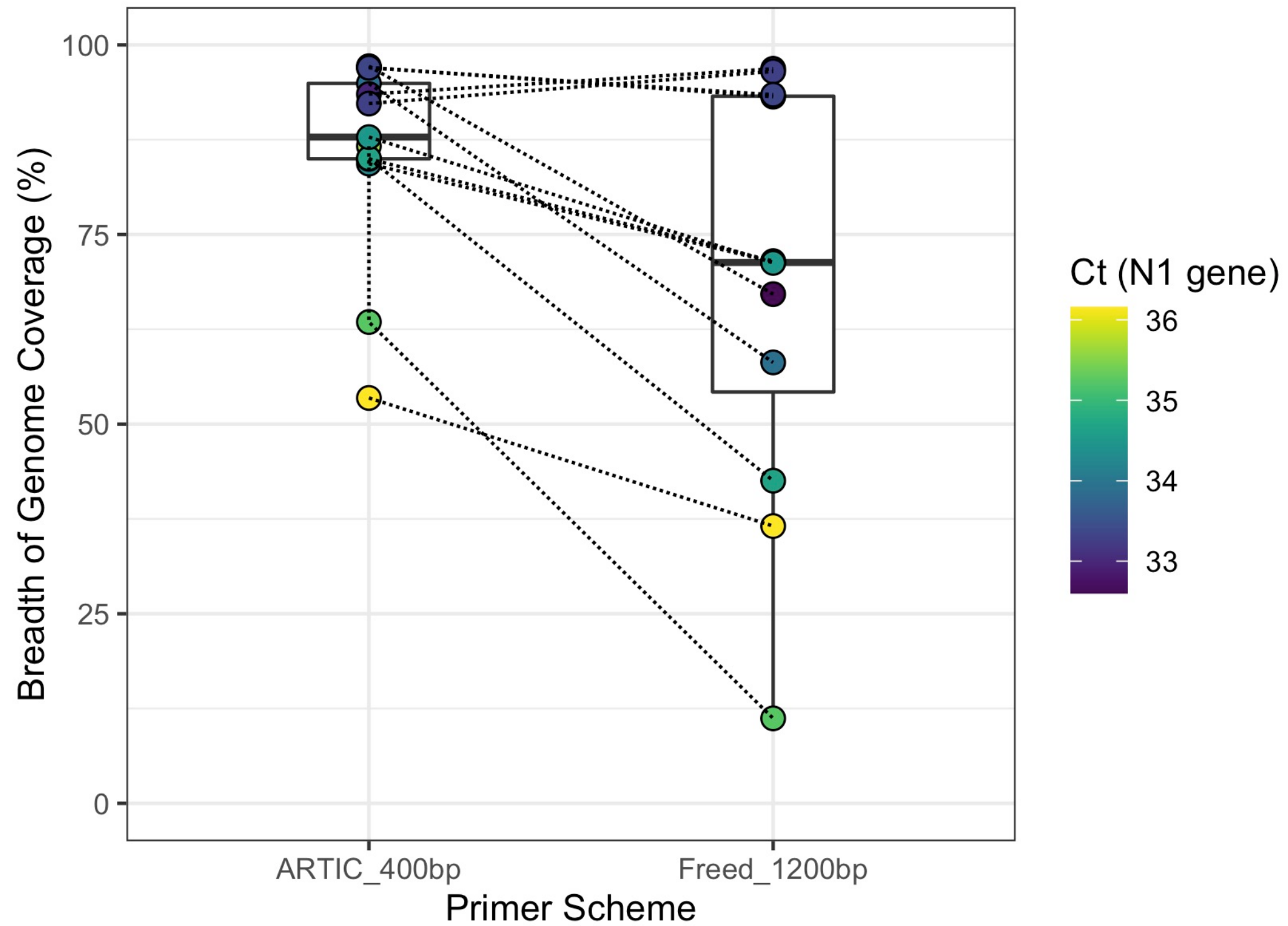

Supplement: FIG S1 [file msystems.01068-21-sf001.pdf]

Breadth of Genome Coverage (%)

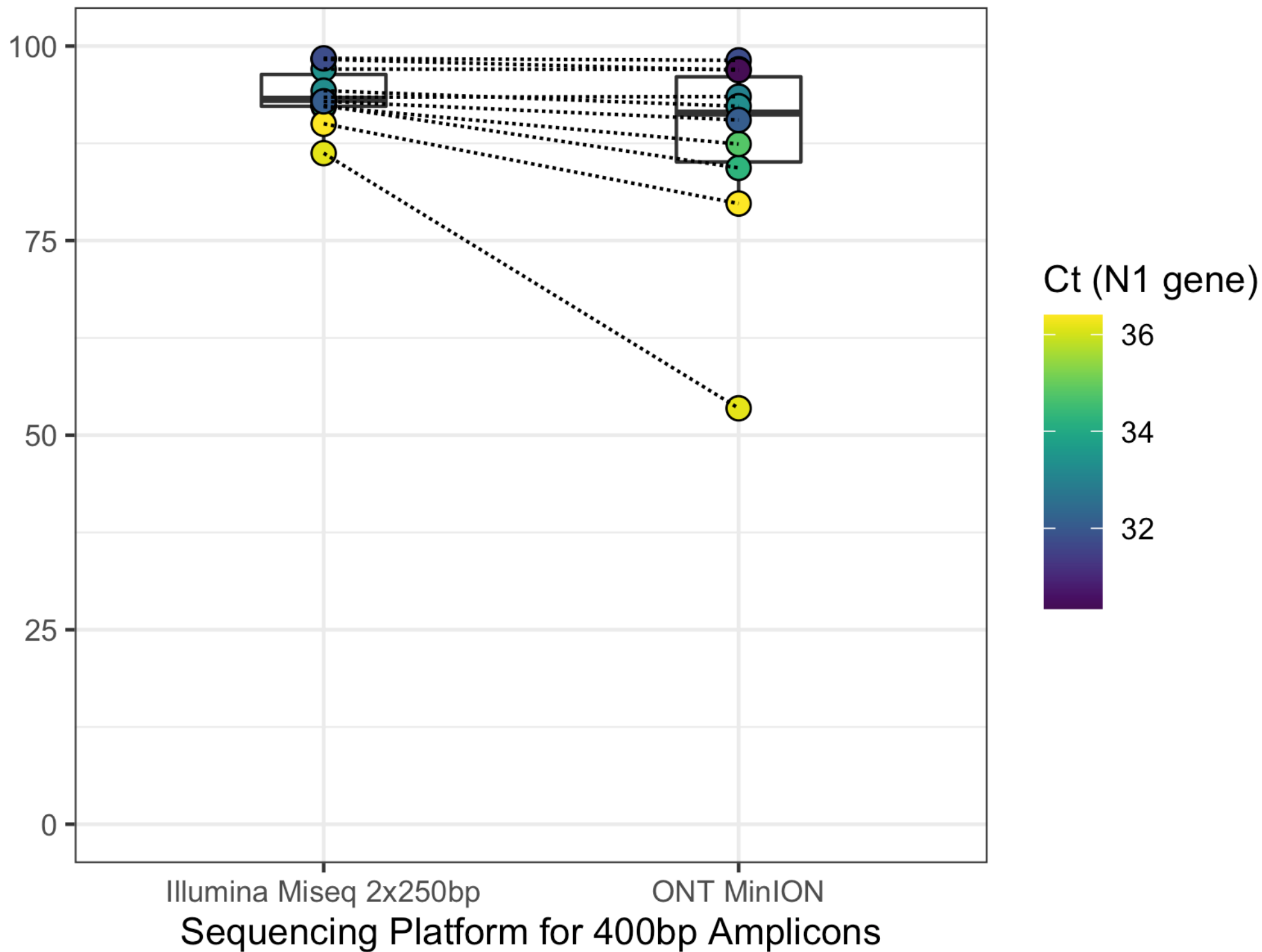

Supplement: FIG S2 [file msystems.01068-21-sf002.pdf]

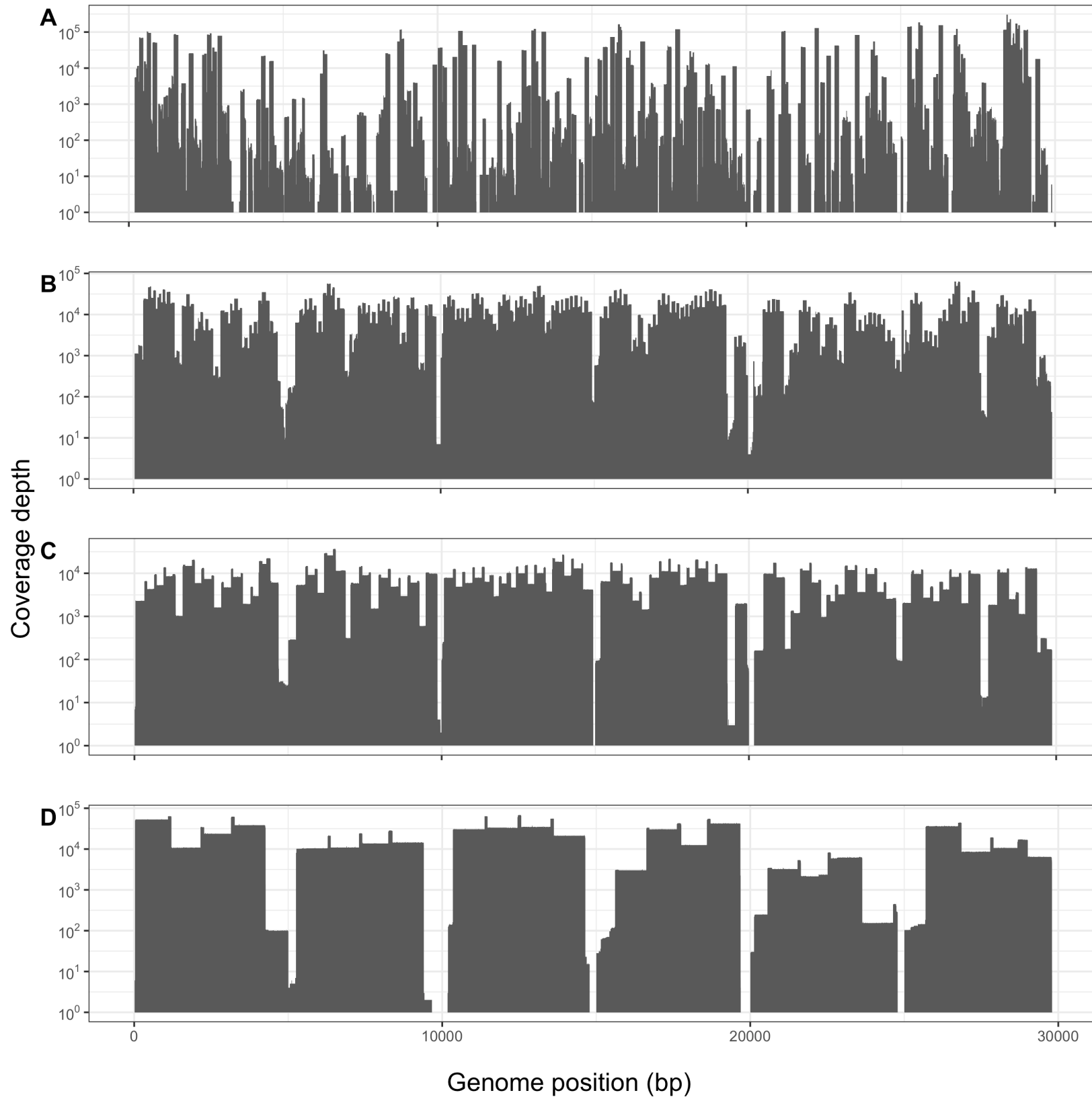

Supplement: FIG S3 [file msystems.01068-21-sf003.pdf]

Mutation

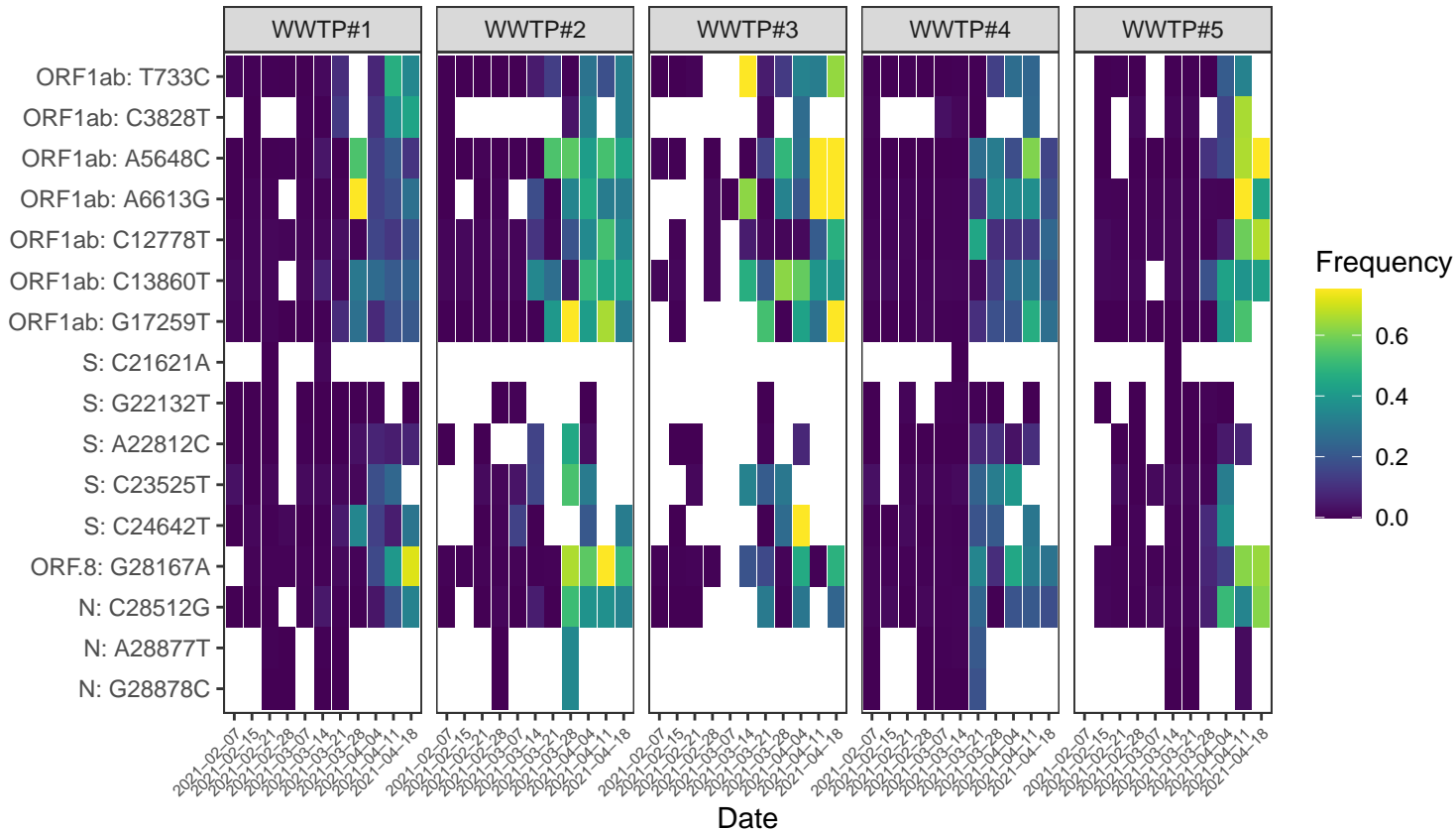

Supplement: FIG S4 [file msystems.01068-21-sf004.pdf]

**A**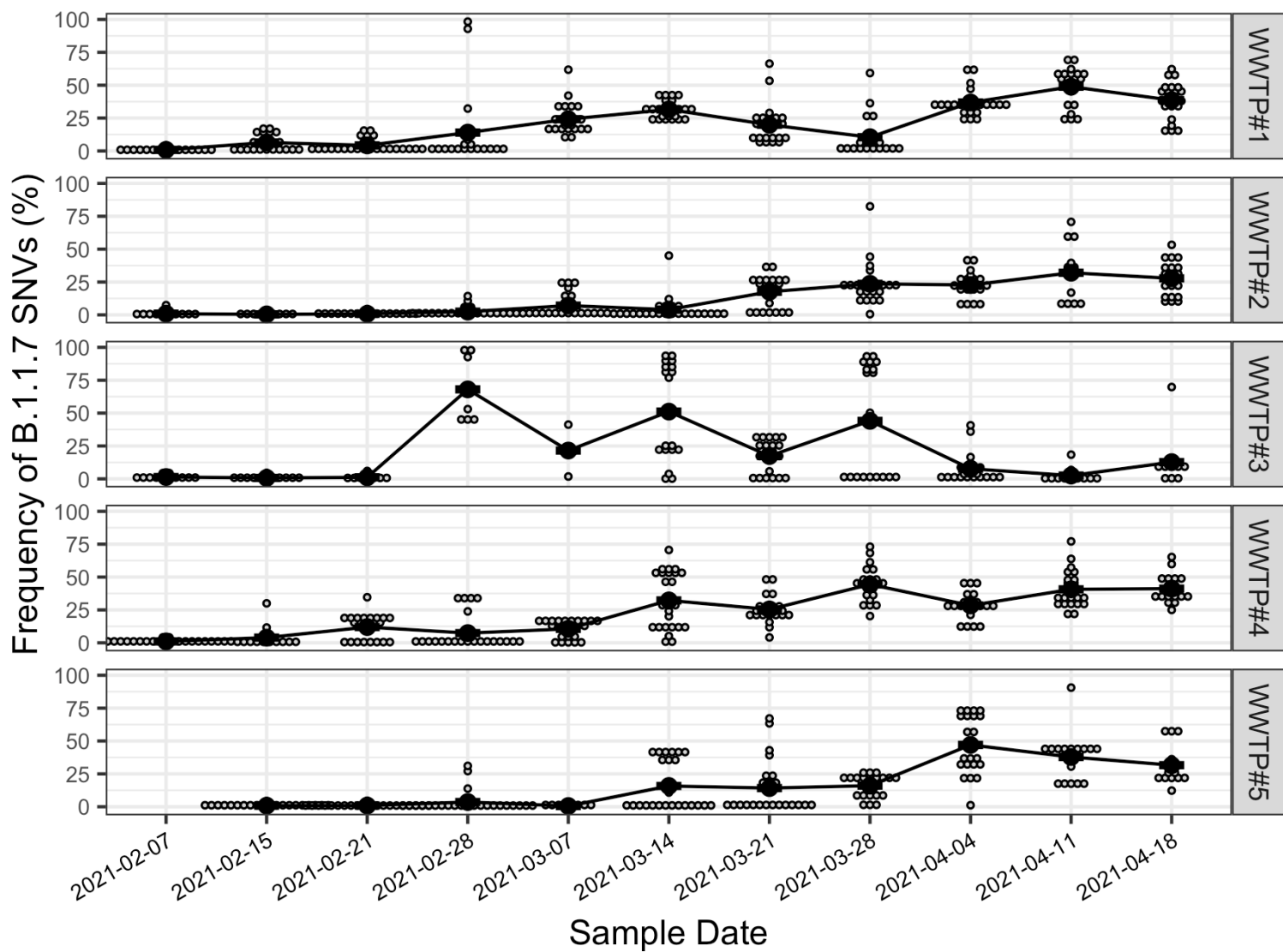**B**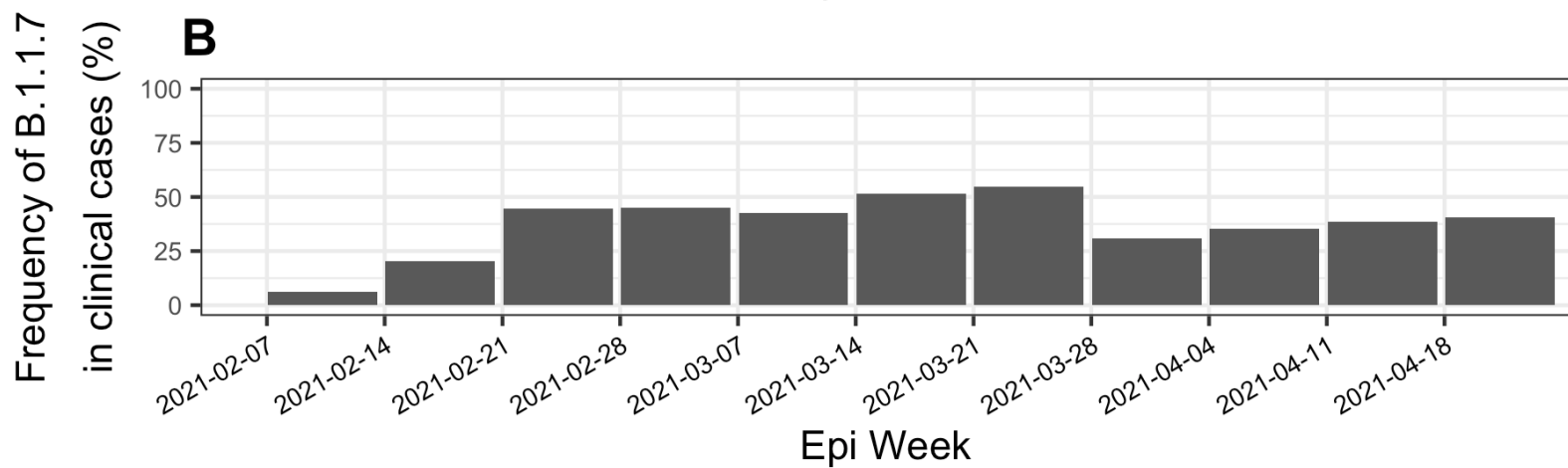**C**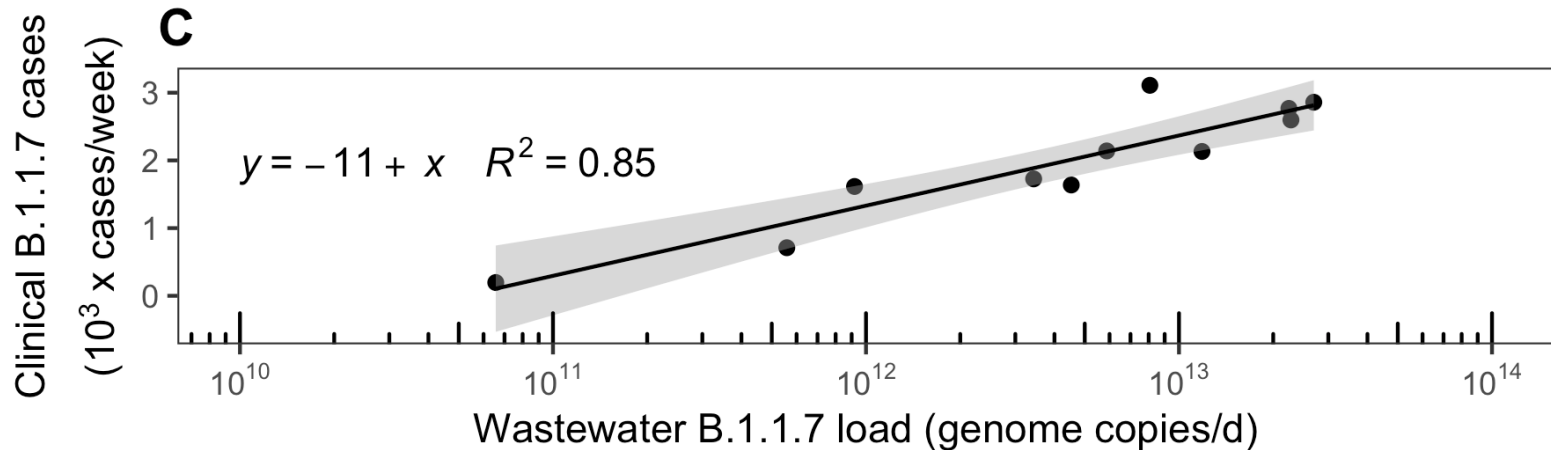

Supplement: FIG S5 [file msystems.01068-21-sf005.pdf]

Mutation

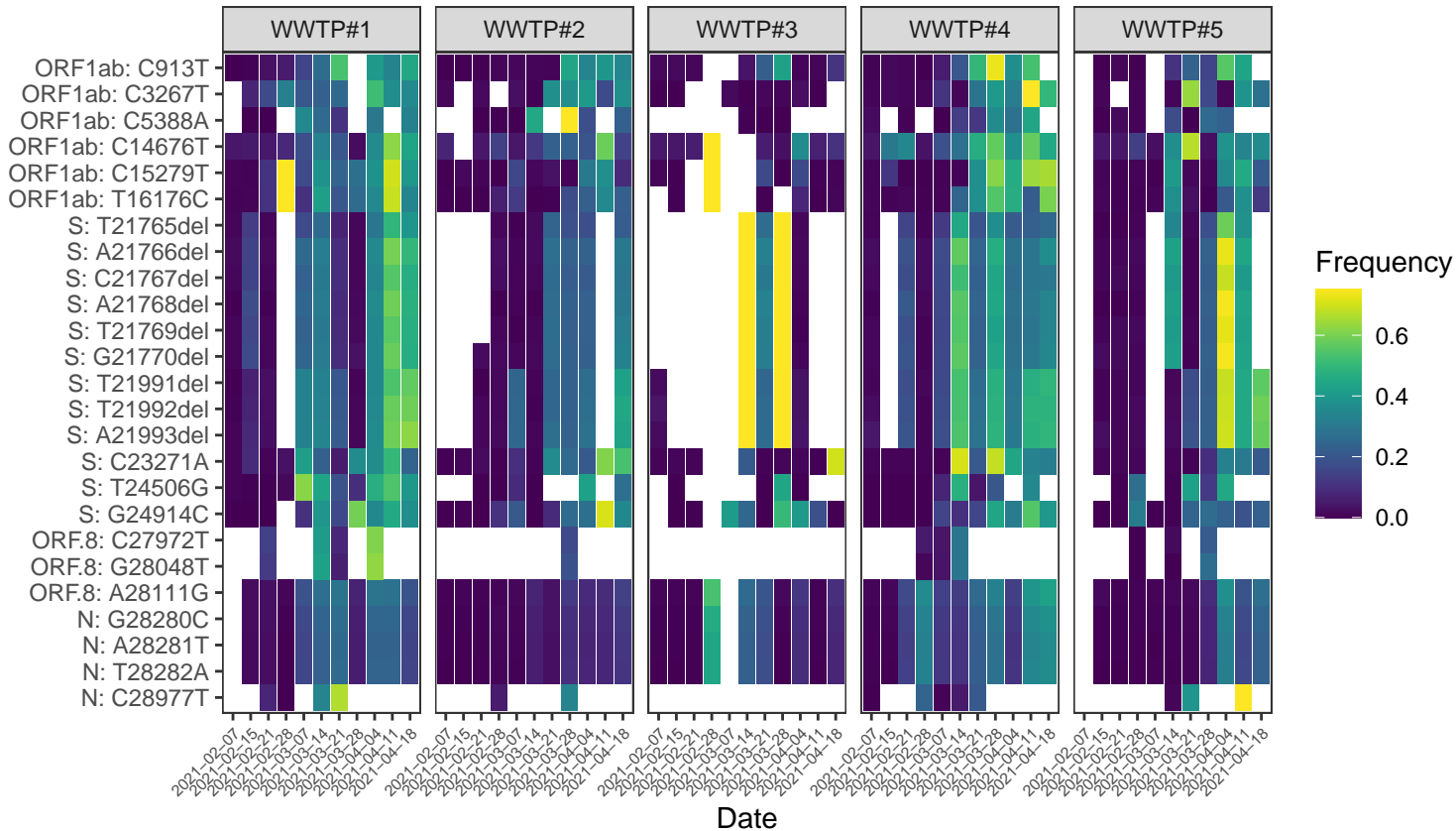

Supplement: FIG S6 [file msystems.01068-21-sf006.pdf]

**A**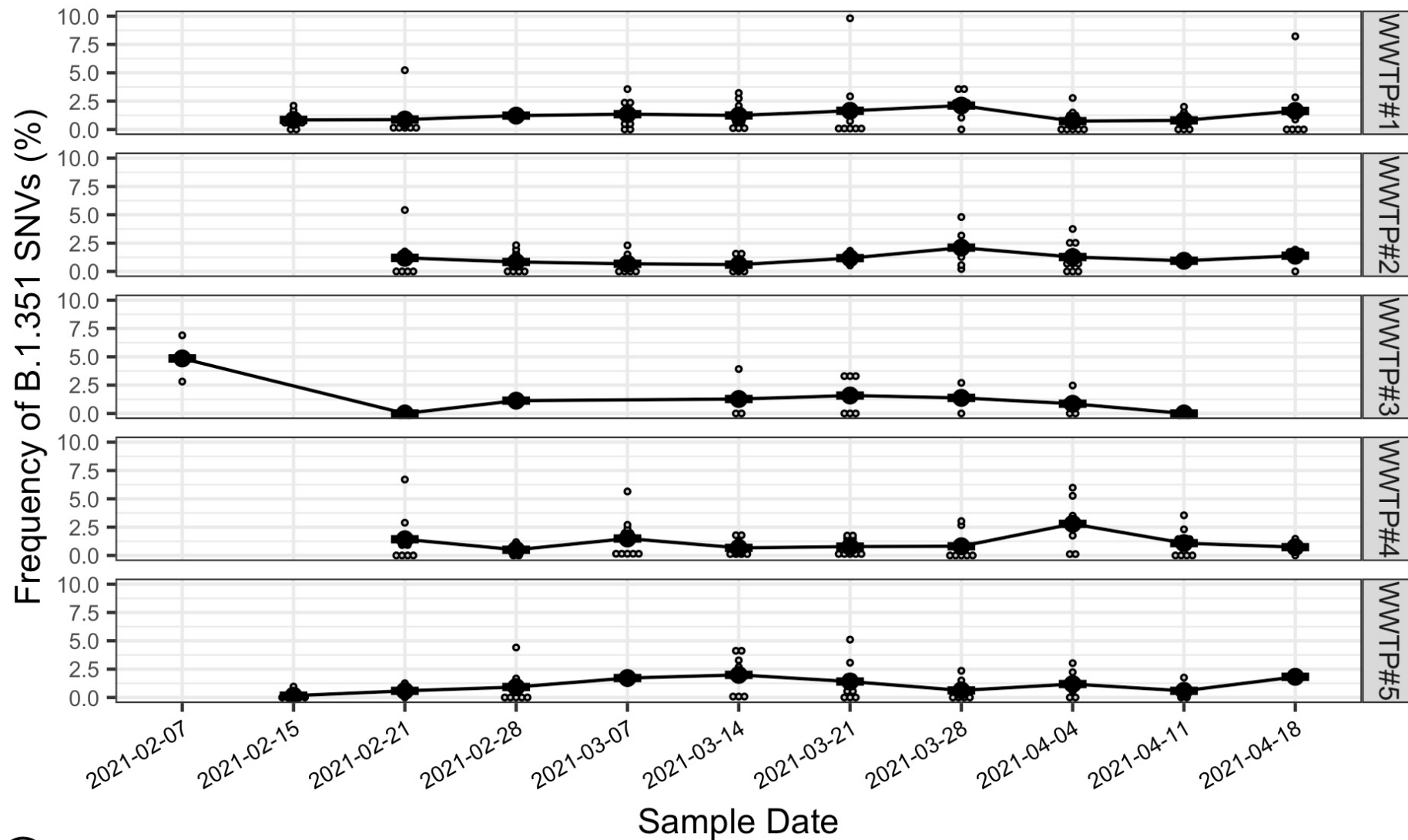

Frequency of B.1.351  
in clinical cases (%)

**B**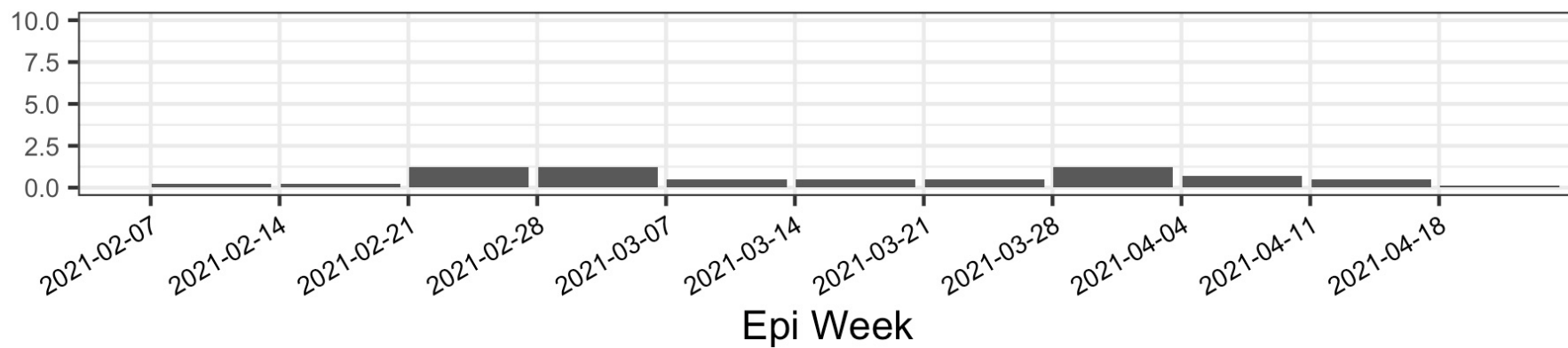

Supplement: FIG S7 [file msystems.01068-21-sf007.pdf]
