# Supplementary material for: Assessing Multiplex Tiling PCR Sequencing Approaches for Detecting Genomic Variants of SARS-CoV-2 in Municipal Wastewater
Source: mSystems. 2021 Oct 19;6(5):e01068-21. doi: 10.1128/mSystems.01068-21 (PMC8525555; doi:10.1128/mSystems.01068-21)
Supplement: FIG S8 [file msystems.01068-21-sf008.pdf]

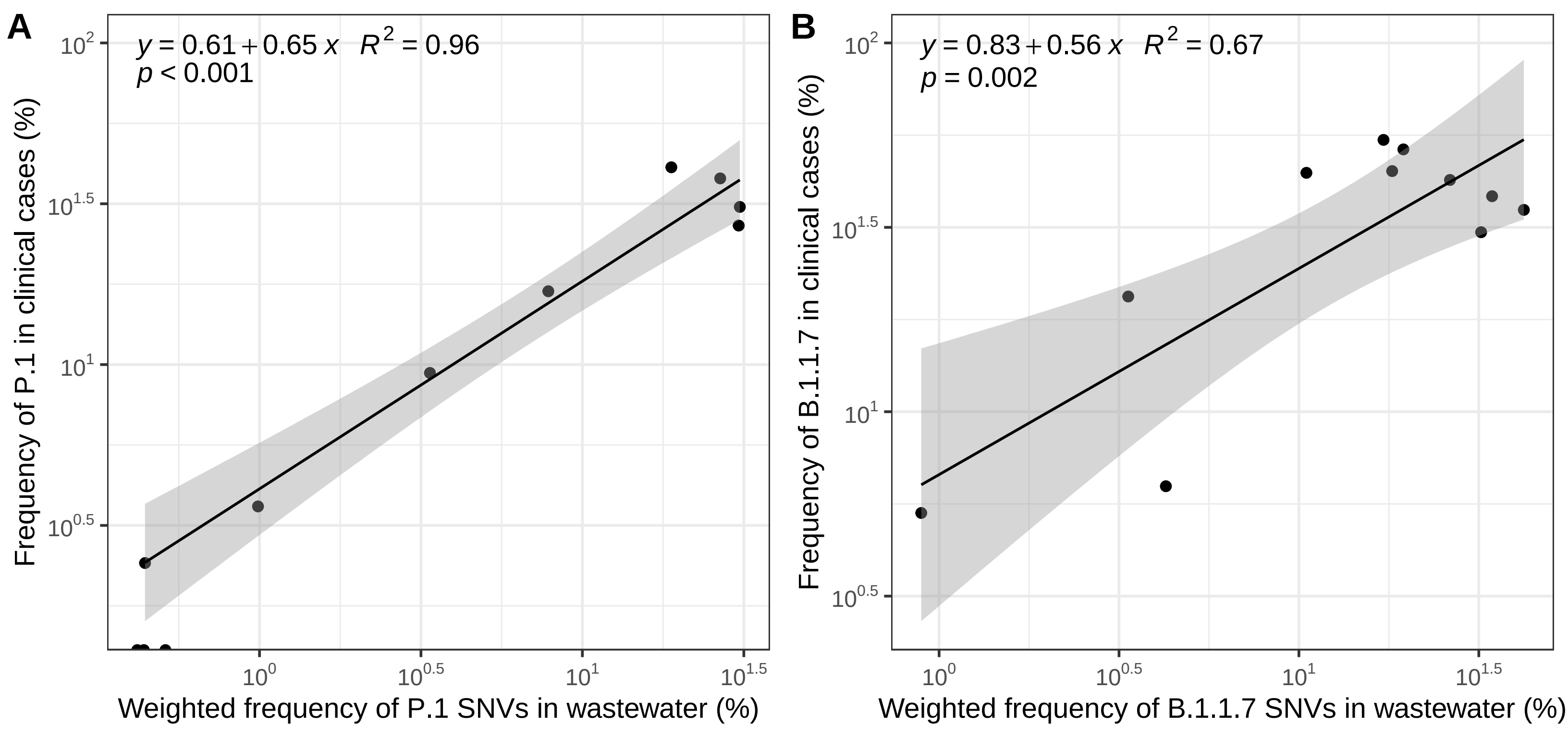
A
$y = 0.61 + 0.65\,x \quad R^2 = 0.96$
$p < 0.001$
Frequency of P.1 in clinical cases (%)
Weighted frequency of P.1 SNVs in wastewater (%)
10^2
$10^{1.5}$
10^1
$10^{0.5}$
10^0
B
$y = 0.83 + 0.56\,x \quad R^2 = 0.67$
$p = 0.002$
Frequency of B.1.1.7 in clinical cases (%)
Weighted frequency of B.1.1.7 SNVs in wastewater (%)
